# Synthesis of Silver Nanoparticles-Modified Graphitic Carbon Nitride Nanosheets for Highly Efficient Photocatalytic Hydrogen Peroxide Evolution

**DOI:** 10.3390/molecules27175535

**Published:** 2022-08-28

**Authors:** Jixiang Hou, Xu Zhang, Kaiwen Wang, Peijie Ma, Hanwen Hu, Xiyuan Zhou, Kun Zheng

**Affiliations:** Beijing Key Laboratory of Microstructure and Properties of Solids, Faculty of Materials and Manufacturing, Beijing University of Technology, Beijing 100124, China

**Keywords:** carbon nitride, hydrogen peroxide, microstructure manipulation, metal nanoparticle modification, photocatalyst

## Abstract

As a promising metal-free photocatalyst, graphitic carbon nitride (g-C_3_N_4_) is still limited by insufficient visible light absorption and rapid recombination of photogenerated carriers, resulting in low photocatalytic activity. Here, we adjusted the microstructure of the pristine bulk-g-C_3_N_4_ (PCN) and further loaded silver (Ag) nanoparticles. Abundant Ag nanoparticles were grown on the thin-layer g-C_3_N_4_ nanosheets (CNNS), and the Ag nanoparticles decorated g-C_3_N_4_ nanosheets (Ag@CNNS) were successfully synthesized. The thin-layer nanosheet-like structure was not only beneficial for the loading of Ag nanoparticles but also for the adsorption and activation of reactants via exposing more active sites. Moreover, the surface plasmon resonance (SPR) effect induced by Ag nanoparticles enhanced the absorption of visible light by narrowing the band gap of the substrate. Meanwhile, the composite band structure effectively promoted the separation and transfer of carriers. Benefiting from these merits, the Ag@CNNS reached a superior hydrogen peroxide (H_2_O_2_) yield of 120.53 μmol/g/h under visible light irradiation in pure water (about 8.0 times higher than that of PCN), significantly surpassing most previous reports. The design method of manipulating the microstructure of the catalyst combined with the modification of metal nanoparticles provides a new idea for the rational development and application of efficient photocatalysts.

## 1. Introduction

Hydrogen peroxide (H_2_O_2_), as one kind of environmentally-friendly chemical [1,2], has attracted widespread interest in a variety of industrial fields, such as environmental remediation, organic synthesis, pulp bleaching, disinfection, and energy transportation [3]. Recently, with the rising awareness of environmental protection and the COVID-19 pandemic, the demand for H_2_O_2_ has continued to grow [4,5]. Oxidation of anthraquinone (AQ) is the most used commercial method for the production of H_2_O_2_, which often requires an energy-intensive process and generates a series of toxic by-products, these deficiencies give impetus to industry and academia together to exploit a sustainable alternative synthesis approach [6,7]. Potocatalytic H_2_O_2_ production only needs semiconductors as photocatalysts, earth-abundant water and molecular oxygen as raw materials [8,9,10], which has attracted much attention as a green, economical and sustainable method. The 2e^−^ water oxidation reaction (2e^−^ WOR) (Equation (1)) and the 2e^−^ oxygen reduction reaction (2e^−^ ORR) (Equation (2)) are two effective ways to realize photocatalytic H_2_O_2_ production [11,12]. In contrast with the 2e^−^ WOR, the 2e^−^ ORR is easier to implement due to the lower thermodynamics (0.695 V versus normalized hydrogen electrode, NHE) [13], that is, enhancing the light-driven 2e^−^ ORR is crucial to achieving the artificial photosynthesis of H_2_O_2_.

2H_2_O → H_2_O_2_ + 2H^+^ + 2e^−^ (1.76 V versus NHE), (1)

O_2_ + 2H^+^ + 2e^−^ → H_2_O_2_ (0.695 V versus NHE), (2)

Among the numerous promising photocatalysts, graphitic carbon nitride (g-C_3_N_4_) possesses a suitable band structure and stable physicochemical properties, enabling the artificial photosynthesis of H_2_O_2_ via the 2e^−^ ORR driven under visible light irradiation [14,15]. However, pristine bulk-g-C_3_N_4_ suffers from the low surface area, insufficient harvesting of visible light, and the high recombination rate of photogenerated carriers, it has remained a challenging task to realize efficient photocatalytic activity [16,17].Until now, various strategies have been employed to address the above deficiencies, such as manipulating nanostructures [18,19], introducing defects [20,21], doping metal/metal-free elements [22], loading single/dual atoms [23,24], nanoclusters [25], and nanoparticles [26], as well as constructing heterojunctions [27,28]. 

Supporting metal nanoparticles, especially noble metal nanoparticles, which exhibit unique intrinsic properties and catalytic activity, have recently emerged as a new frontier in photocatalysis research [29,30]. Noble metal nanoparticles possess a surface plasmon resonance (SPR) effect located in the visible light range, which significantly enhances the visible light adsorption capacity [31,32]. Moreover, the Schottky barrier formed at the interface of noble metal nanoparticles and the semiconductor photocatalyst can capture photogenerated electrons, suppressing the recombination of photogenerated electron-hole pairs [33]. Supported noble metal photocatalysts, such as platinum (Pt), gold (Au), and palladium (Pd), have been reported in recent years to improve the photocatalytic selectivity and activity of pristine bulk-g-C_3_N_4_. For example, Zuo et al. [34] carried out a study on the g-C_3_N_4_ supported Au nanoparticles as an efficient photocatalyst for H_2_O_2_ production under visible light irradiation. The Au/C_3_N_4_ exhibited remarkably enhanced photocatalytic activity than pristine bulk-g-C_3_N_4_, which can be attributed to the efficient separation of charge carriers between the finely dispersed Au cocatalyst and C_3_N_4_. Similarly, Ding et al. [35] reported a low-temperature inductively coupled plasma technique to grow Pt nanoparticles onto the pristine bulk-g-C_3_N_4_. As a result, the absorption in the visible light region was enhanced, while the recombination efficiency of the charge carriers was suppressed. However, most of the research has focused on the metal nanoparticles-modification of pristine bulk-g-C_3_N_4_, which is lack of exposed surfaces, resulting in insufficient loading of metal nanoparticles. Recent research has demonstrated that exfoliating and cleaving out g-C_3_N_4_ nanosheets via thermal etching or ultrasonic etching is an effective method to significantly increase the specific surface area and porosity of bulk-g-C_3_N_4_. This thin-layer, nanosheet-like structure is beneficial for the loading of metal nanoparticles, while exposing more active sites for the adsorption and activation of reactants. Besides, the reduction of the interlayer size effectively improves the charge transfer efficiency [20,36], which is beneficial to accelerate the process of photocatalytic reaction. 

**Figure 1:**
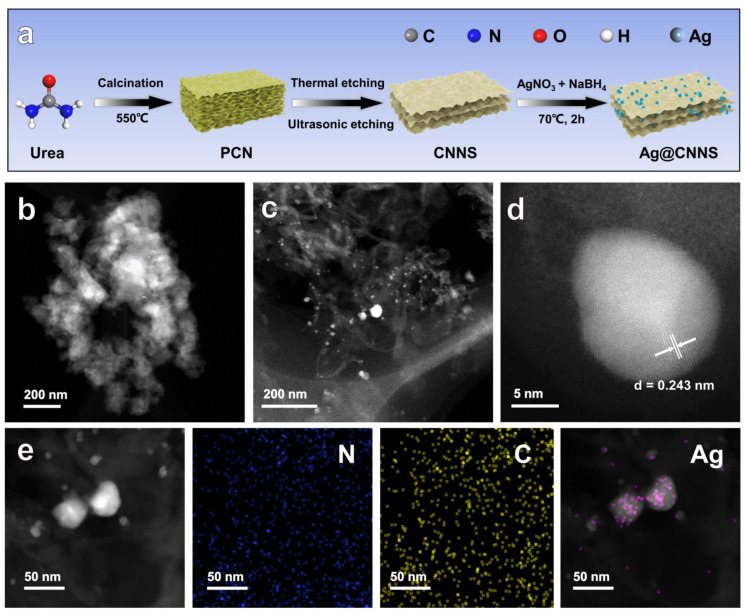
(**a**) Schematic illustration of the preparation process of Ag@CNNS; TEM images of (**b**) PCN and (**c**) Ag@CNNS; (**d**) AC-HAADF-STEM image of Ag nanoparticles loaded on the CNNS; (**e**) HAADF-STEM image and corresponding EDS elemental mapping images of Ag@CNNS.

Although Teng et al. [13] proposed a single Sb atom dispersed on carbon nitride (Sb-SAPC), which achieved an extremely high photocatalytic H_2_O_2_ generation rate in a phosphate buffer solution (without any sacrificial agent), the H_2_O_2_ yield in pure water still needs to be improved. Herein, by tuning the microstructure of pristine bulk-g-C_3_N_4_ (PCN), thin-layer porous g-C_3_N_4_ nanosheet (CNNS) was designed and synthesized via a continuous thermal etching and ultrasonic etching method. Considering that silver (Ag) is a noble metal with low price and high crustal abundance [37,38], with suitable catalytic properties and optical properties [39], Ag nanoparticles were successfully grown on the CNNS by chemical reduction to facilitate Ag nanoparticles-modified g-C_3_N_4_ nanosheets (Ag@CNNS). The preparation process of Ag@CNNS could be schematically illustrated in Figure 1a. Subsequently, the mechanism of the enhanced photocatalytic performance of PCN was systematically investigated. The presence of Ag nanoparticles enhanced both the visible light absorption ability and the separation capability of photogenerated carriers, making a superior photocatalytic activity for H_2_O_2_ evolution (120.53 μmol/g/h) under visible light irradiation. The unique properties of Ag@CNNS accelerate the efficiency of the whole artificial photocatalytic H_2_O_2_ production.

## 2. Experimental Section

### 2.1. Materials

Urea (CH_4_N_2_O, 99%), sodium borohydride (NaBH_4_, 98.0%), potassium iodide (KI, ≥99.99%), potassium phthalate monobasic (C_8_H_5_O_4_K, 99.8%), silver nitrate solution (AgNO_3_, 0.1000 mol/L) and ethanol (EtOH, ≥99.5%) were all purchased from Shanghai Aladdin Biochemical Technology Co., Ltd., Shanghai, China. All the chemical reagents were used without further purification. Deionized water was used throughout the entire experiment.

### 2.2. Preparation of Samples

#### 2.2.1. Preparation of PCN and CNNS

PCN was synthesized using a conventional thermal polymerization method [40]. 15 g of urea was placed in a 100 mL alumina crucible with a lid and calcined at 550 °C with a ramping rate of 5 °C/min in a muffle furnace. After being cooled to room temperature naturally, the obtained yellow solids were collected and milled into a powder. Finally, washed these products several times with deionized water and ethanol, and dried at 80 °C for 12 h.

CNNS was synthesized using thermal etching and ultrasonic etching in sequence. Briefly, 150 mg of the as-prepared PCN was placed in a tube furnace and heated to 550 °C under an argon atmosphere. During the heating process, the size of the yellow powder gradually decreased, resulting in a yellowish solid. Subsequently, collected the product and dispersed in 40 mL of deionized water by ultrasonication for 12 h. The resulting solution was centrifuged to recover CNNS, which was then washed, and dried at 80 °C for 12 h.

#### 2.2.2. Preparation of Ag@CNNS

Ag@CNNS was synthesized using a chemical reduction method. In detail, 60 mg of CNNS was placed into 30 mL of deionized water and ultrasonicated for 30 min to achieve uniform dispersion, followed by the addition of 30 mL of ethanol. Then, added with 0.6 mL of AgNO_3_ solution (0.1000 mol/L) and 2 mL of NaBH_4_ solution (0.2 mg/mL) sequentially, and kept stirring at 70 °C for 2 h. Finally, the obtained products were washed with deionized water and ethanol several times, respectively, and dried at 80 °C for 12 h.

### 2.3. Characterization

Transmission electron microscope (TEM) images were collected using an FEI Titan Themis G2 microscope operated at an accelerating voltage of 300 kV. The powder X-ray diffraction (XRD) patterns of the samples were collected on a Bruker, D8 ADVANCE with Cu K_α_ radiation (λ = 0.15418 nm) at room temperature. The specific surface areas of all samples were measured according to the Brunner-Emmet-Teller (BET) method on a BELSORP-max II. The UV–vis absorption spectra (UV-vis) and UV-vis diffuse reflectance spectroscopy (UV-DRS) of the samples were carried out on a HITACHI U-3900H spectrophotometer (wavelength range of 300–700 nm) and HITACHI UH4150 spectrophotometer (with BaSO_4_ as reference material), respectively. Photoluminescence (PL) spectra were measured on a Hitachi F-7000 fluorescence spectrometer with an excitation wavelength of 375 nm. 

### 2.4. Photocatalytic Evaluation

The photocatalytic H_2_O_2_ production by the as-prepared catalysts was evaluated using a 300 W Xe lamp (Beijing Perfect Light, PLS-SXE300C) without the absence of any sacrificial agent. Here, 25 mg of each photocatalyst was added to 25 mL of deionized water (1 mg/mL) in a specific reactor. Ultrasonic treatment for 30 min to make the powder completely dispersed, and then O_2_ was bubbled through the solution to make the O_2_ saturated. Next, maintain the reaction solution at 25 °C under a circulating water system and keep the photocatalyst dispersed under magnetic stirring, while irradiating the reaction solution with 400 nm cutoff film (λ ≥ 400 nm) to start the photoreaction test. During the photoreaction progress, 1.5 mL of the reaction solution was taken out to remove the photocatalyst every 15 min. 

The concentrations of H_2_O_2_ were measured by iodometry [41]. 0.5 mL of 0.1 mol/L C_8_H_5_KO_4_ aqueous solutions and 0.5 mL of 0.4 mol/L KI aqueous solution were added to 1 mL of the obtained supernatant and then kept for 60 min. The following reaction occurs in the mixed solution: H_2_O_2_ + 3I^−^ + 2H^+^ → I^3−^ + 2H_2_O, the generated I^3^^−^ has a strong absorption peak around 350 nm, from which the amount of H_2_O_2_ produced during the photocatalytic reaction can be calculated by UV–vis spectroscopy. Figure S1 shows the standard curve of H_2_O_2_ and the UV-vis absorption intensity of different H_2_O_2_ concentrations by iodimetry. 

### 2.5. Electrochemical Tests

The electrochemical impedance spectroscopy (EIS) and Mott–Schottky plots were performed on an electrochemical workstation (CHI600A) using a three-electrode setup with ITO glasses covered by the as-prepared photocatalysts as working electrodes, a Pt wire as the counter electrode, and Ag/AgCl electrode as the reference electrode. The conversion relationship between the measured potentials (vs. Ag/AgCl) and reversible hydrogen electrode (RHE) at 25 °C is as follows (Equation (3)):

E_RHE_ = E_Ag/AgCl_ + 0.0591 pH + 0.197, (3)

## 3. Results and Discussion

### 3.1. Microscopic Morphology and Structural Characterization of PCN, CNNS and Ag@CNNS

Transmission electron microscopy (TEM) was performed to observe the structure and morphology of as-prepared samples. Figure 1b and Figure S2 (Supplementary Materials) showed low magnified TEM images. As can be seen in Figure 1b, PCN presented a typical agglomerated bulk-like structure without obvious pores. In contrast, the thermal etching and ultrasonic etching significantly altered the morphology as can be seen in Figure S2 (Supplementary Materials). The obtained CNNS exhibited a loose, porous nanosheet-like structure with larger specific surface area and more abundant pore structure (Figure S4, Supplementary Materials). The special structure which not only provided a large number of active sites for reactant adsorption and activation but also shortened the transport distance of photogenerated carriers [36]. Using NaBH_4_ to reduce Ag(Ⅰ) into Ag nanoparticles, which can be observed in the TEM images. Figure 1c and Figure S3 (Supplementary Materials) clearly showed some bright spots corresponding to the monodisperse, spherical-like Ag nanoparticles uniformly distributed on the CNNS, with a size of around 10 nm. Moreover, Ag@CNNS also exhibited a nanosheet-like structure. The photocatalyst was also analyzed using Scanning TEM (STEM) coupled with energy-dispersive spectroscopy (EDS) to investigate the crystal structure and elemental distribution, respectively. From the aberration-corrected high-angle annular dark-field STEM (AC-HAADF-STEM) image (Figure 1d), it could be seen that the projection of Ag nanoparticles in top view was approximately circular. The clear lattice fringes with an interplane distance of 0.243 nm could be attributed to the (111) lattice space of Ag [30]. Furthermore, the EDS mapping of Ag@CNNS (Figure 1e) further confirmed that the compound is composed of C, N, and Ag elements, indicating the homogeneous distribution of Ag nanoparticles on CNNS. TEM images combined with the N_2_ physisorption measurements (Figure S4 and Table S1, Supplementary Materials) showed that the microscopic structure of the PCN was precisely controlled, exhibiting a thin-layer nanosheet-like structure, and the Ag nanoparticles were uniformly distributed on the substrate. 

The microstructure of the photocatalysts was further investigated via powder X-ray diffraction (XRD). As shown in Figure S5 (Supplementary Materials), Both PCN and Ag@CNNS exhibited two diffraction peaks at 13.4° and 27.3°, corresponding to the (100) and (002) planes, which could be attributed to the in-plane structure of tri-s-triazine units and the stacking of the conjugated aromatic system, respectively [42]. Although the presence of Ag nanoparticles could be confirmed by TEM images, no diffraction peaks of Ag nanoparticles were observed in the XRD pattern due to the extremely low Ag content [43]. 

### 3.2. Optical Properties and Band Structures of PCN, CNNS and Ag@CNNS

**Figure 2:**
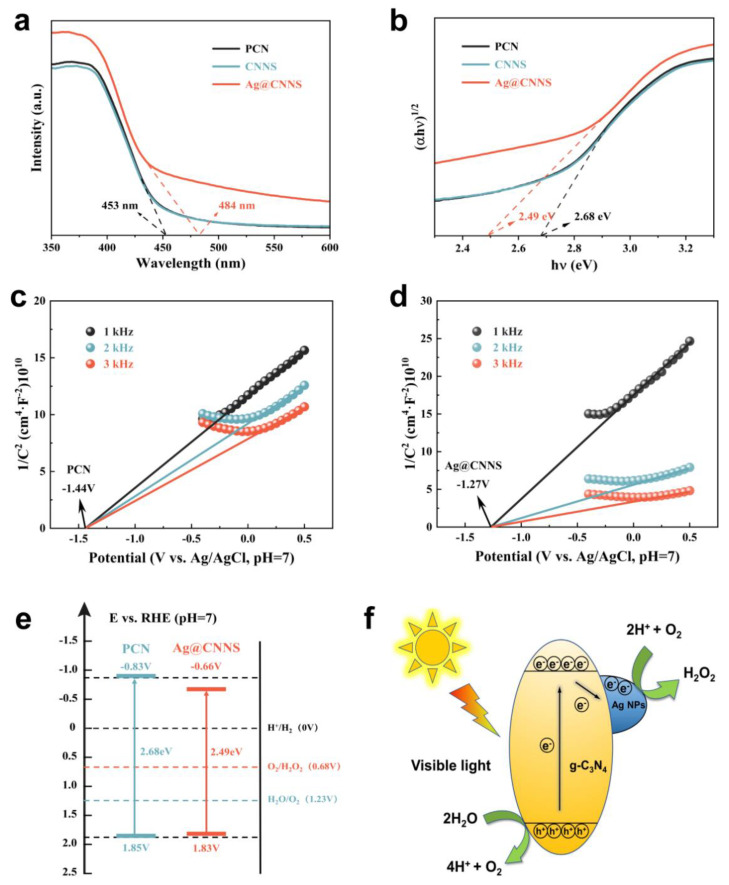
(**a**) UV-vis DRS and (**b**) plots of transformed Kubelka-Munk function versus photon energy of PCN, CNNS, and Ag@CNNS; Mott-Schottky plots of (**c**) PCN, and (**d**) AgCNNS; (**e**) the schematic diagram of the band structure of PCN and Ag@CNNS; (**f**) schematic illustration of the enhanced photocatalytic mechanism of Ag@CNNS under visible-light irradiation.

Sunlight provides the initial driving force for the entire artificial photosynthesis process, which is closely related to the optical property and band structure of the photocatalyst. Optimizing the mentioned above factors is an essential prerequisite for promoting photocatalytic activity. Notably, the modification of the microstructure, as well as the introduction of noble metal nanoparticles can significantly affect the optical property and band structure of the photocatalyst. Using UV-vis diffuse reflectance spectra (DRS) to reveal the optical absorption properties of the samples. As shown in Figure 2a, PCN had an absorption edge of about 453 nm in the visible light region, the thermal etching and ultrasonic etching process only had a weak impact on the light absorption of PCN. Especially, after the introduction of Ag nanoparticles, the light absorption intensity was obviously enhanced as well as the optical absorption edge had a significant red shift compared with the other two photocatalysts, which indicated that more photogenerated electrons would participate in the photocatalytic process. This phenomenon could be attributed to the SPR effect of Ag nanoparticles, which had a signature optical performance [31,44]. Besides, according to the transformed Kubelka-Munk function (Figure 2b), the band gaps of the samples changed significantly, narrowing from 2.68 eV to 2.49 eV, suggesting that Ag-related midgap states are generated and altered the band structure of PCN. To clarify the reason for the narrowing of the band gap in Ag@CNNS, Mott-Schottky analysis was measured on the ITO (Figure 2c,d). The conduction band (CB) potential (versus the Ag/AgCl, pH = 7) of Ag@CNNS was changed from −1.44 V (for PCN) to −1.27 V. Combined with the bandgap values calculated by the Kubelka-Munk function, the band structure of the samples was estimated as shown in Figure 2e. The comparison suggested that the introduction of Ag nanoparticles was unaffected on the valance band (VB) potential, on the contrary, the position of the CB was markedly lower, making the band structure of Ag@CNNS more suitable for the activation of O_2_ on the surface of photocatalyst (Figure 2f).

### 3.3. Photogenerated Carriers Transfer Behavior of PCN, CNNS and Ag@CNNS

**Figure 3:**
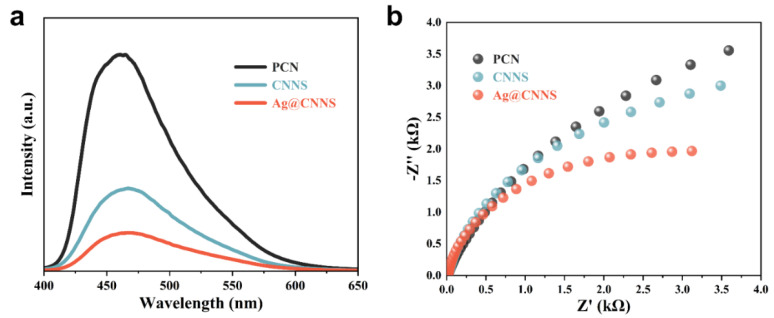
(**a**) PL spectra and (**b**) EIS Nyquist plots of PCN, CNNS, and Ag@CNNS.

To systematically investigate the separation, transfer, and recombination behavior of photogenerated carriers during the catalytic progress, steady-state photoluminescence (PL) and electrochemical impedance spectroscopy (EIS) were carried out. As shown in Figure 3a, all the samples showed an emission peak at about 465 nm with a different emission intensity. Evidently, in contrast with PCN, CNNS exhibited a lower emission intensity, since the decrease of the microscopic size reduced the migration distance of photogenerated carriers to the surface of the catalyst [33,45]. Despite the excellent optical properties of Ag@CNNS, the emission intensity was the lowest, which indicated that the introduction of Ag nanoparticles inhibited the rapid recombination of photogenerated electron-hole pairs and realized the efficient separation and transfer of photogenerated carriers [34]. Similar results could be further confirmed by EIS (Figure 3b). By fitting the EIS Nyquist plots, the semicircle diameter of Ag@CNNS was significantly smaller than that of others, which indicated that Ag@CNNS possessed the smallest charge transfer resistance, thus facilitating the transport of photogenerated carriers. The contact between Ag nanoparticles and CNNS formed a Schottky barrier, which could capture photogenerated electrons, and significantly improved the separation and transport efficiency of photogenerated electron-hole pairs [32].

### 3.4. Photocatalytic H_2_O_2_ Production of PCN, CNNS and Ag@CNNS

**Figure 4:**
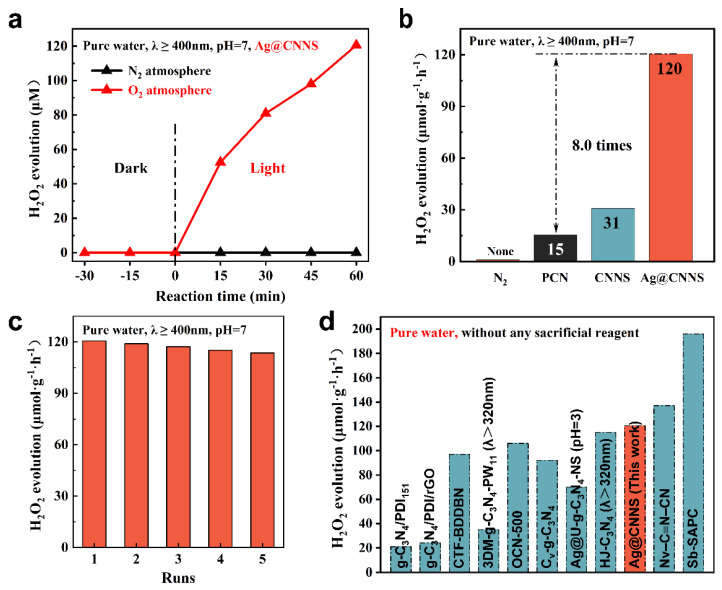
(**a**) H_2_O_2_ yields of different times during the photocatalytic reaction; (**b**) the photocatalytic H_2_O_2_ generation rates of different samples under visible-light irradiation; (**c**) cycling test of photocatalytic H_2_O_2_ production over Ag@CNNS; (**d**) a comparison of photocatalytic H_2_O_2_ production between Ag@CNNS and other g-C_3_N_4_-based photocatalysts in recent years.

The photocatalytic production of H_2_O_2_ by PCN, CNNS, and Ag@CNNS was further evaluated in pure water (without any sacrificial agent). Figure 4a,b summarized the photocatalytic H_2_O_2_ yields of various catalysts during the evaluation process. As shown in Figure 4b, the photocatalytic activity of CNNS was greatly enhanced, which indicated that the loose and porous nanosheet-like structure facilitated the adsorption of reactants and the transfer of photogenerated carriers on the surface of the photocatalyst [21,46]. Furthermore, the Ag@CNNS exhibited an unparalleled photocatalytic performance, with the H_2_O_2_ generation rate reaching 120.53 μmol/g/h, about 8.0 times higher than that of PCN (15.40 μmol/g/h). It could be interpreted as follows: (1) The nanosheet-like structure exposed more surface for reactant adsorption and activation. (2) The SPR effect of Ag nanoparticles narrowed the band gap and promoted the absorption of visible light. (3) A Schottky barrier was formed at the interface between Ag nanoparticles and the substrate to capture the photogenerated electrons, thus realizing the effective separation of photogenerated electron-hole pairs. Considering the stability in practical applications, a cycling test has been carried out. As shown in Figure 4c, after 5 cycles of photocatalytic tests, no significant decrease in the H_2_O_2_ yield could be observed. Ag nanoparticles were still uniformly and firmly dispersed on the g-C_3_N_4_ nanosheets (Figure S6, Supplementary Materials), indicating the excellent stability of the interface between Ag nanoparticles and the substrate. TEM image of the cycled Ag@CNNS combined with the cycling test of photocatalytic H_2_O_2_ production fully confirmed that Ag@CNNS has excellent cyclability and stability. Furthermore, the Ag@CNNS was also evaluated under a dark condition and nitrogen atmosphere, respectively. No H_2_O_2_ could be detected, suggesting the importance of visible light and O_2_ in the photoredox progress [40]. Finally, we summarized the performance comparison of g-C_3_N_4_-based photocatalysts for H_2_O_2_ production in recent years (Figure 4d and Table 1). Based on previous related work, it can be considered that Ag@CNNS is a highly active photocatalyst for H_2_O_2_ production.

## 4. Conclusions

In summary, we successfully optimized the optical properties, band structure and carrier transfer behavior of the pristine bulk-g-C_3_N_4_ via microstructure control and noble metal nanoparticle modification. The introduction of Ag nanoparticles not only enhanced the light absorption properties but also realized the effective capture of photogenerated electrons. Besides, the thin-layer nanosheet-like structure not only accelerated the transfer of charges but also promoted the adsorption and activation of reactants. As a result, the photocatalytic H_2_O_2_ yield of the resulted Ag@CNNS reached 120.53 μmol/g/h, about 8.0 times higher than that of PCN, significantly improving photocatalytic activity. This work provides a promising guide for the rational design of efficient g-C_3_N_4_-based photocatalysts for H_2_O_2_ production.

## Figures and Tables

**Table 1:** Summary of the photocatalytic production of H_2_O_2_ with g-C_3_N_4_-based photocatalysts.

Photocatalysts	Concentration of Photocatalyst (mg/mL)	Reaction Solution	H_2_O_2_ Yields (μmol)	Ref.
Ag@CNNS (This work)	1.00	pure water (pH = 7)	120.53 (1 h)	-
Nv-C≡N-CN	1.00	pure water (pH = 7)	137 (1 h)	[40]
HJ-C_3_N_4_	1.00	pure water (pH = 7)	115 (1 h)	[27]
ZnPPc-NBCN	0.50	pure water (pH = 7)	57 (1 h)	[47]
OCN-500	1.00	pure water (pH = 7)	53 (10 h)	[48]
PEI/C_3_N_4_	1.00	pure water (pH = 7)	208.1 (AM 1.5 G, 1 h)	[49]
Co_1_/AQ/C_3_N_4_	0.50	pure water (pH = 7)	62 (AM 1.5 G, 1 h)	[46]
g-C_3_N_4_/PDI_51_	1.67	pure water (pH = 7)	31 (24 h)	[15]
g-C_3_N_4_/BDI_51_	1.67	pure water (pH = 7)	41 (24 h)	[15]
Ag@U-g-C_3_N_4_-NS	1.00	pure water (pH = 3)	70 (1 h)	[30]
Sb-SAPC15	2.00	Phosphate buffer solution	470.5 (8 h)	[13]
g-C_3_N_4_	4.00	90% ethanol	30 (12 h)	[50]
g-C_3_N_4_	1.67	10% isopropanol	148 (6 h)	[14]
NDCN	1.00	10% isopropanol	476 (1 h)	[51]
TC/pCN	1.00	10% isopropanol	131.71 (1 h)	[28]
CN_4_	0.50	10% isopropanol	287 (1 h)	[19]

## Data Availability

Not applicable.

## Supplementary Materials

The following supporting information can be downloaded at: https://www.mdpi.com/article/10.3390/molecules27175535/s1, Figure S1: the standard curve of H_2_O_2_ and the UV-vis absorption intensity of different H_2_O_2_ concentrations by iodimetry; Figure S2: the low magnified TEM images of CNNS; Figure S3: the low magnified TEM images of Ag@CNNS; Figure S4: (a) N_2_ adsorption-desorption isotherms and (b) pore size distribution curves of PCN, CNNS, and Ag@CNNS; Figure S5: XRD patterns of PCN and Ag@CNNS; Figure S6: TEM image of Ag@CNNS after a 5 times cycling test; Figure S7: (a) schematic diagram of the Ag@CNNS; (b) local coordination structure of Ag nanoparticles; Table S1: BET surface areas of PCN, CNNS, and Ag@CNNS.

## Sample Availability

Samples of the compounds are available from the authors.
