# Supplementary material for: Synthesis of Silver Nanoparticles-Modified Graphitic Carbon Nitride Nanosheets for Highly Efficient Photocatalytic Hydrogen Peroxide Evolution
Source: Molecules. 2022 Aug 28;27(17):5535. doi: 10.3390/molecules27175535 (PMC9457636; doi:10.3390/molecules27175535)
Supplement: Supplementary file 1 [file molecules-27-05535-s001.zip › molecules-1867175-supplementary.pdf]

molecules

MDPI

Supplementary Material

Synthesis of Silver Nanoparticles-Modified Graphitic Carbon Nitride Nanosheets for Highly Efficient Photocatalytic Hydrogen Peroxide Evolution

Jixiang Hou †, Xu Zhang †, Kaiwen Wang, Peijie Ma, Hanwen Hu, Xiyuan Zhou, and Kun Zheng *

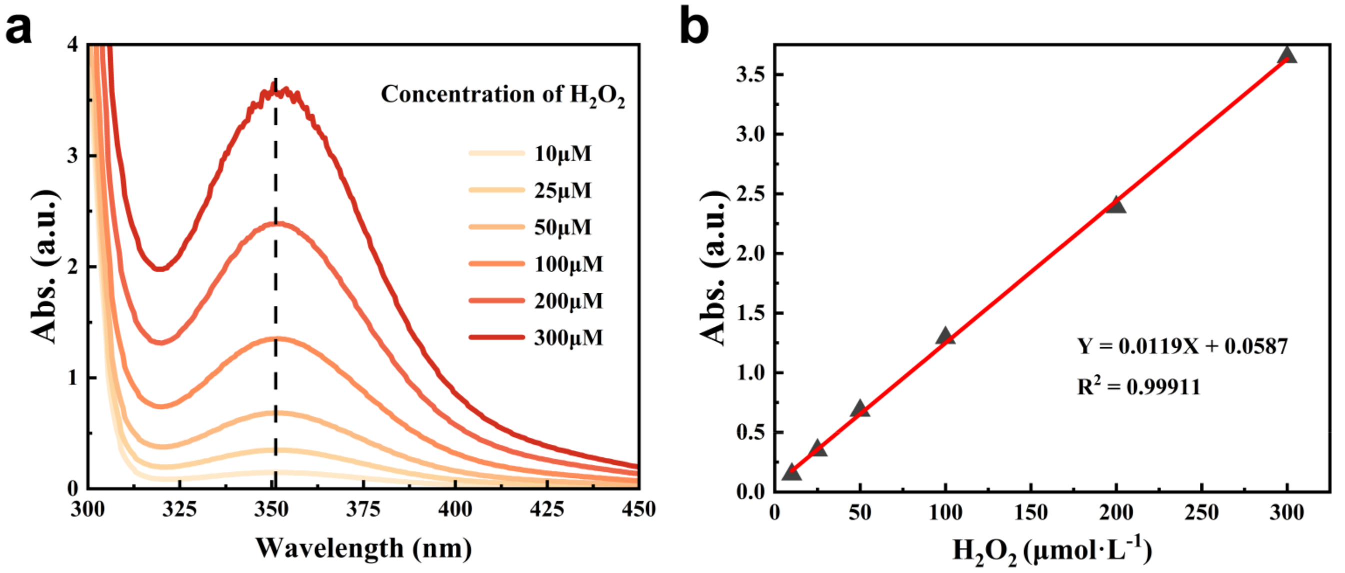

Figure S1. (a) the UV-vis absorption intensity of different H_2O_2 concentrations by iodimetry; (b) the standard curve of H_2O_2.

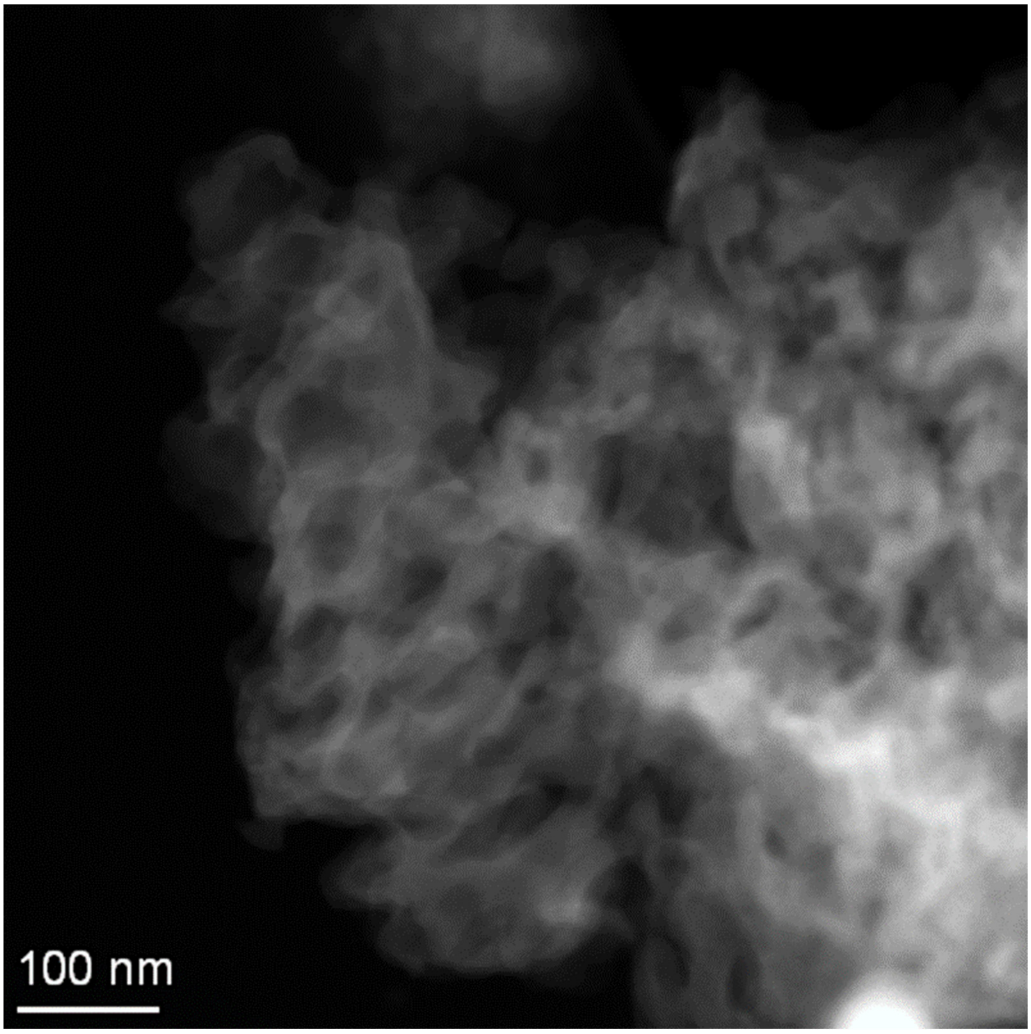

Figure S2. the low magnified TEM image of CNNS.

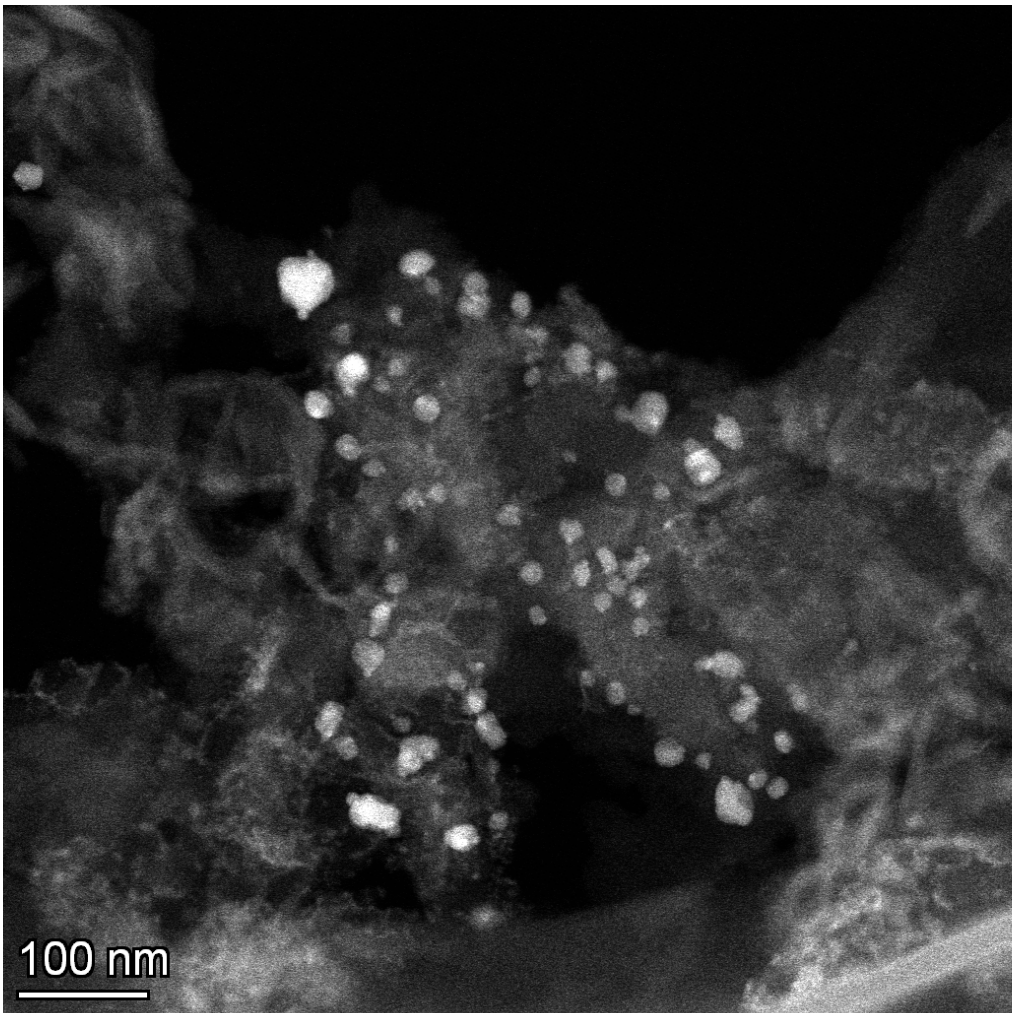

Figure S3. the low magnified TEM image of Ag@CNNS.

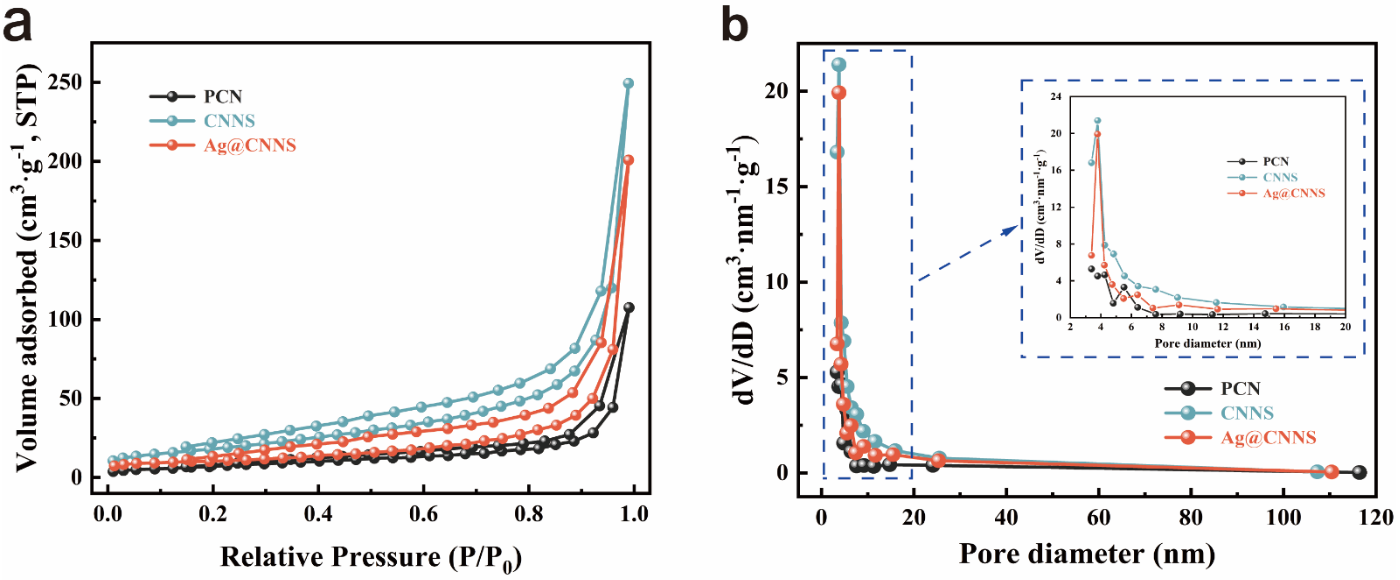

Figure S4. (a) N_2 adsorption-desorption isotherms and (b) pore size distribution curves of PCN, CNNS, and Ag@CNNS.

Table S1. BET surface areas of PCN, CNNS, and Ag@CNNS.

Photocatalysts	BET surface area ($m^2 \cdot g^{-1}$)
PCN	27.836
CNNS	67.716
Ag@CNNS	37.736

As shown in Figure S4a, all samples exhibited a type IV N_2 adsorption-desorption isotherm and a type H3 hysteresis loop. Table S1 further summarized the specific surface area of the samples in the following order: CNNS (67.716 m^2/g) > Ag@CNNS (37.736 m^2/g) > PCN (27.836 m^2/g). The CNNS had the largest specific surface area, indicating that PCN was successfully exfoliated into g-C_3N_4 nanosheets by thermal etching and ultrasonic etching. Compared with CNNS, the specific surface area of Ag@CNNS was slightly reduced, because Ag nanoparticles on the surface blocked pores of CNNS, which also reflected the effective loading of Ag particles on CNNS. Meanwhile, Figure S4b further showed the pore size distribution of the samples. In contrast, CNNS and Ag@CNNS had a more wider pore size distribution than PCN, presenting a pore size distribution curve centered at 4 nm.

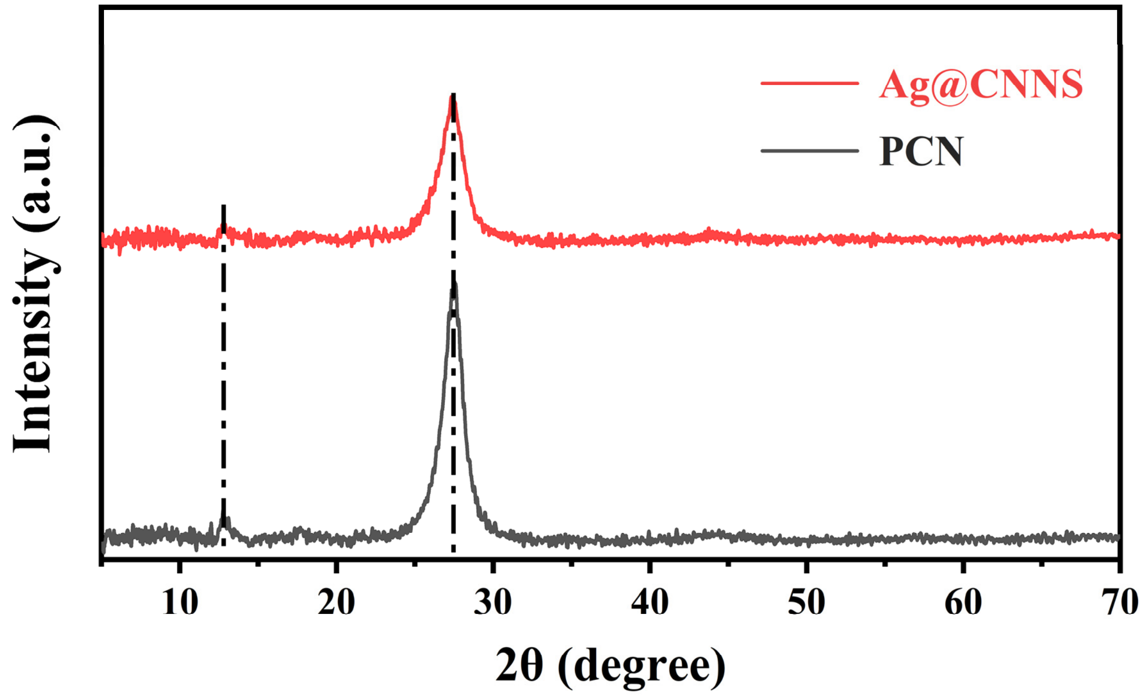

Figure S5. XRD patterns of PCN and Ag@CNNS.

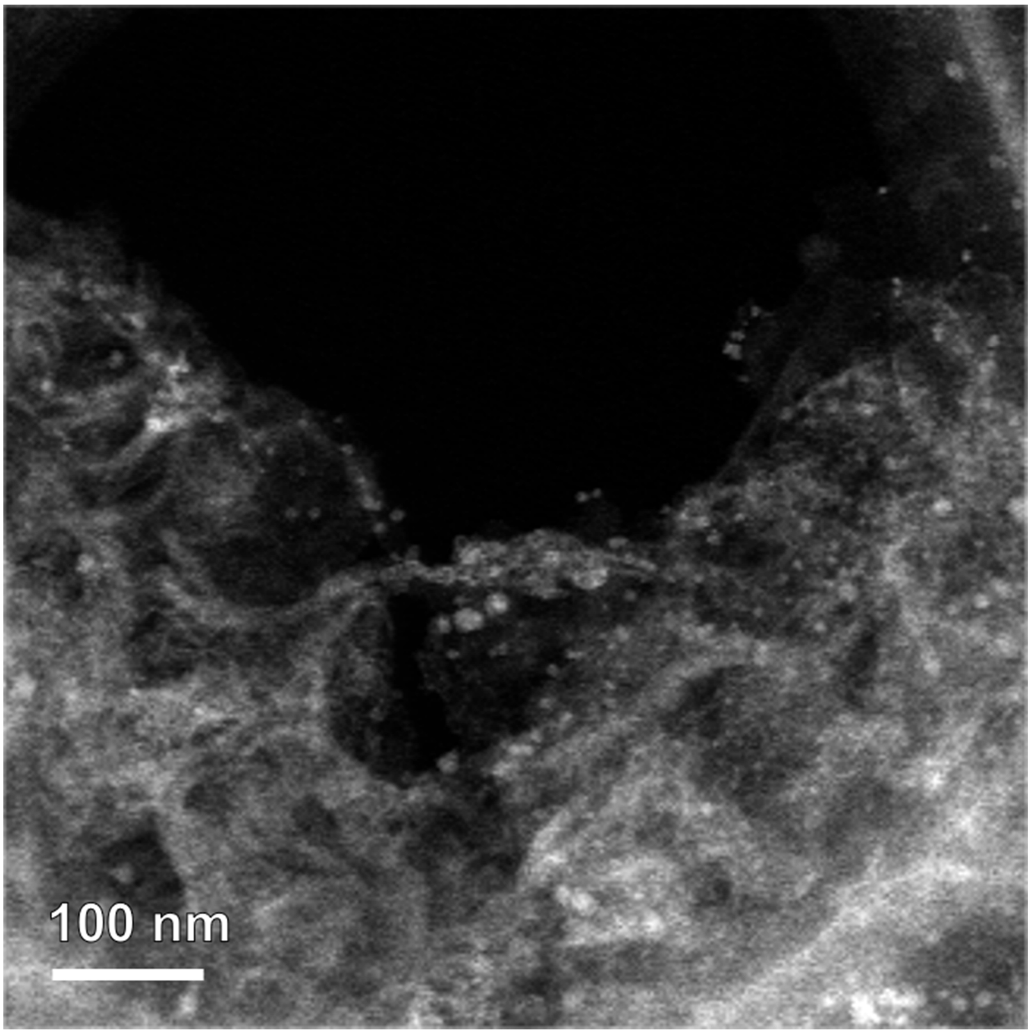

Figure S6. TEM image of Ag@CNNS after a 5 times cycling test.

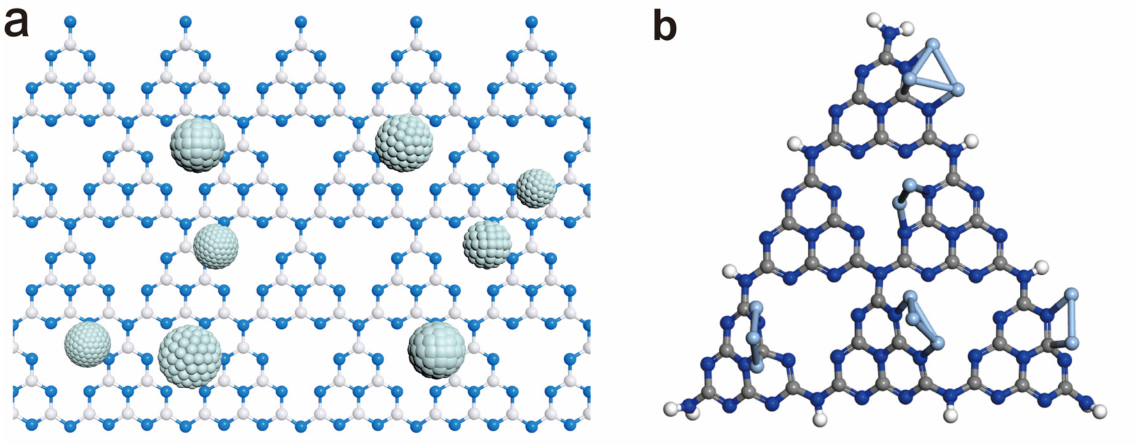

Figure S7. (a) schematic diagram of the Ag@CNNS; (b) local coordination structure of Ag nanoparticles.
